# A vision-based closed-loop framework for adaptive ergonomic human-robot collaboration

**DOI:** 10.3389/frobt.2026.1948863

**Published:** 2026-10-14

**Authors:** Sakol Teeravarunyou, Supachai Vongbunyong, Kochahem Kamolwit, Pongsak Kitirojpan, Chayanat Lertwittayanuruk

**Affiliations:** 1 Human-Centered Design LAB, School of Architecture and Design, King Mongkut’s University of Technology Thonburi, Bangkok, Thailand; 2 Institute of Field Robotics, King Mongkut’s University of Technology Thonburi, Bangkok, Thailand

**Keywords:** closed-loop adaptation, collaborative robots, computer vision, ergonomics, human-centered robotics, human-robot collaboration

## Abstract

**Introduction:**

Although collaborative robots can safely share workspaces with human operators, most collaborative workstations remain static and cannot adapt to changes in operator posture during task execution, potentially compromising ergonomics and interaction quality. This study presents a vision-guided closed-loop adaptive control framework that enables a collaborative robot to continuously adjust workpiece position according to the operator’s posture.

**Methods:**

Using real-time vision-based human pose estimation, the system automatically modifies workpiece height and reach distance to support ergonomic working postures. The framework was evaluated with 30 participants performing screw-driving tasks under fixed and adaptive workstation conditions.

**Results:**

Compared with the fixed workstation, the adaptive workstation significantly reduced Rapid Upper Limb Assessment scores, improved physical comfort, reduced perceived postural load, and increased trust and acceptance without compromising perceived safety. In addition, it reduced the influence of participant height on ergonomic performance, demonstrating improved anthropometric adaptability.

**Discussion:**

These findings demonstrate that real-time posture perception can serve as an effective feedback mechanism for adaptive robot control, providing a practical framework for human-centered collaborative workstations that accommodate operator variability while improving ergonomic outcomes and user experience.

## 1 Introduction

Collaborative robots (cobots) are increasingly deployed in manufacturing environments to work alongside human operators, supporting the transition toward more flexible and human-centered production systems (Villani et al., 2018; Lorenzini et al., 2023). Unlike conventional industrial robots that execute predefined motions, cobots operate in shared workspaces where human behavior and task conditions continuously change. Consequently, effective human–robot collaboration depends not only on safe physical interaction but also on the robot’s ability to perceive human behavior and adapt its actions accordingly. However, many collaborative workstations remain statically configured, requiring operators to adjust their posture to the workstation rather than allowing the workstation to adapt to individual workers. This limitation highlights the need for adaptive collaborative workstations capable of responding dynamically to changes in human posture (Ojsteršek et al., 2024).

Among the various sources of information available for adaptive human–robot collaboration, body posture provides a direct representation of the operator’s physical interaction with the workspace. During manual assembly, posture continuously changes in response to workstation geometry, task demands, and individual anthropometric characteristics. Prolonged awkward upper-limb postures and excessive reaching have been consistently associated with increased physical workload and work-related musculoskeletal disorders (Das and Sengupta, 1996; Yang et al., 2020). Consequently, monitoring posture enables collaborative robots to adapt workstation configurations according to individual operator needs rather than relying on fixed ergonomic assumptions, supporting more personalized and human-centered collaboration (Deutsches Institut für Normung, 2005; Boschetti et al., 2022).

Recent advances in computer vision have enabled continuous human posture estimation without requiring intrusive wearable sensors. Vision-based posture estimation techniques have demonstrated sufficient accuracy for monitoring upper-limb kinematics in industrial environments while providing practical sensing solutions for collaborative robotics (Kim et al., 2023; Lafayette et al., 2023). Among the available postural variables, elbow flexion was selected as the control variable because it is an important postural variable in widely used ergonomic assessment tools such as RULA (McAtamney and Corlett, 1993), is sensitive to workstation height, reach distance, and workpiece orientation, and can be reliably estimated using vision-based skeletal tracking (Kim et al., 2023; Lafayette et al., 2023). These characteristics make elbow flexion a practical feedback variable for closed-loop robot adaptation, enabling the robot to adjust its position in response to the user’s posture. While elbow flexion was used as the control variable for workstation adaptation, overall ergonomic performance was evaluated using RULA, which incorporates multiple postural factors to provide a more comprehensive assessment of ergonomic risk (McAtamney and Corlett, 1993).

Recent studies have incorporated ergonomic information into human–robot collaboration using various approaches. These include workstation adaptation through adjustable workstations (Colim et al., 2021; Martins et al., 2025), posture-based robot motion adaptation (van den Broek and Moeslund, 2020; Meregalli Falerni et al., 2024), and vision-based ergonomic assessment using human pose estimation (Menanno et al., 2024). Optimization-based methods have also been proposed to balance productivity with ergonomic risk during collaborative tasks (Keshvarparast et al., 2024). Collectively, these studies demonstrate the growing importance of integrating ergonomic information into collaborative robotics; however, they typically emphasize robot motion planning, posture assessment, or optimization independently, with ergonomic information used primarily for assessment or predefined adaptation rather than as a real-time feedback signal for continuous, closed-loop workstation adjustment. As a result, relatively few collaborative workstations can continuously convert changes in operator posture into adaptive robot behavior during task execution.

To address these limitations, this study proposes a vision-based closed-loop ergonomic control framework for adaptive human–robot collaboration. The framework continuously estimates upper-limb posture using vision-based posture estimation and employs elbow flexion as the control variable to dynamically adjust workpiece height and reach distance through closed-loop robot control. By integrating human posture perception, ergonomic decision-making, and adaptive robot control within a unified architecture, the proposed framework provides personalized workstation adaptation based on the operator’s posture.

## 2 Proposed framework

### 2.1 Framework overview

The proposed framework enables adaptive human–robot collaboration by continuously perceiving the operator’s upper-limb posture and adapting the collaborative workstation through closed-loop robot control. As illustrated in Figure 1, the framework consists of three interconnected modules: (1) vision-based human posture perception, (2) ergonomic decision-making, and (3) robot adaptation. These modules operate continuously throughout task execution to establish a perception–decision–adaptation feedback loop, enabling the robot to continuously adjust the workpiece configuration according to the operator’s posture rather than executing predefined motions.

**FIGURE 1:**
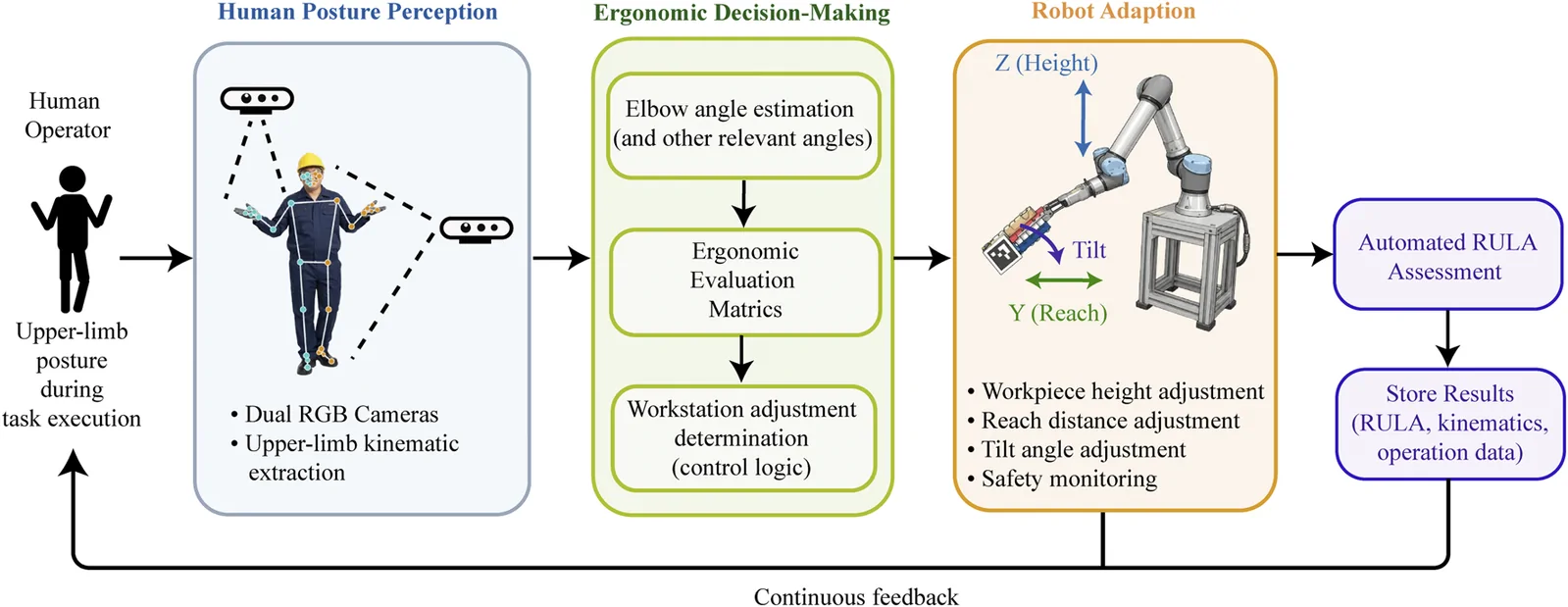
Vision-based closed-loop framework.

During task execution, the vision module continuously estimates the operator’s upper-limb posture using vision-based posture estimation and extracts the kinematic information required for robot adaptation. The estimated posture is transmitted to the ergonomic decision-making module, where it is evaluated against predefined ergonomic criteria to determine whether workstation adjustment is required. When adaptation is necessary, the robot adaptation module generates motion commands to modify the workpiece position and orientation. Following each adjustment, the operator’s posture is re-estimated, allowing the collaborative robot to continuously respond to changes in the operator’s posture throughout task execution.

### 2.2 Vision-based human posture perception

The vision-based human posture perception module continuously estimates the operator’s upper-limb posture during task execution and provides the kinematic information required for adaptive robot behavior. The module functions as the perception layer of the proposed framework by converting the operator’s movements into posture information for the ergonomic decision-making module.

Two independent RGB cameras were employed for distinct posture-tracking purposes. Each camera was an Intel RealSense Depth Camera D435i, operated in RGB mode at a resolution of 1920 × 1080 and a frame rate of 30 fps. A front-facing camera captured the operator’s upper-body posture, while a second, top-mounted camera captured a downward view of the operator’s hand and wrist, since the operator’s body was not visible from this viewpoint. The captured RGB images from both cameras were processed independently using the Google MediaPipe framework (version 0.10.14): the front-facing camera stream was processed with the MediaPipe Pose model to extract shoulder, elbow, and wrist landmarks for upper-body posture estimation, while the top-mounted camera stream was processed with the MediaPipe Hands model to extract hand landmarks for wrist posture assessment. No cross-camera coordinate registration or triangulation was performed; each camera’s landmarks were used exclusively within their respective analysis pipeline. MediaPipe was selected because it provides real-time, markerless human pose estimation (Lugaresi et al., 2019). Markerless pose estimation has been shown to be a feasible approach for ergonomic assessment (Kim et al., 2021), and recent studies have further demonstrated the applicability of MediaPipe for automated ergonomic risk assessment in occupational environments (Singhtaun et al., 2024).

From the front-facing camera’s skeletal output, the shoulder, elbow, and wrist landmarks of the operator’s dominant upper limb were extracted for posture estimation. The upper-arm orientation angle (θ_arm) and lower-arm flexion angle (θ_lower), computed from these landmarks, served as the primary control variables for workstation adaptation (see Section 2.3). Hand landmarks from the top-mounted camera were used separately to assess wrist posture as part of the automated RULA scoring described below.

A pre-experimental validation study was conducted with five participants to assess the accuracy of the vision-based joint-angle estimation. Each participant was positioned at six predefined elbow flexion angles (60°, 75°, 90°, 105°, 120°, and 135°), with the target angles established using a goniometer as the reference measurement. The estimated elbow angles were obtained from the MediaPipe-based vision pipeline and compared with the corresponding goniometer measurements. Across the 30 observations (five participants × six target angles), the system achieved a mean absolute error (MAE) of 6.30° and a root-mean-square error (RMSE) of 7.62°. The estimated and reference angles showed a strong linear association (Pearson’s r = 0.977). The estimates showed a small systematic tendency to underestimate the reference angle, with a mean bias of approximately −5.3°. These results indicate that the vision-based pipeline provided reasonably accurate elbow-angle estimation across the tested range.

### 2.3 Ergonomics decision-making

The ergonomic decision-making module determines the appropriate workstation adjustment based on the operator’s estimated upper-limb posture. The process begins by computing the upper-arm orientation from the shoulder and elbow landmarks detected by the vision system. The vector components are calculated in Equation 1.

$$\left[\begin{array}{c} \Delta x\\ \Delta y \end{array}\right]=\left[\begin{array}{c} -\left(x_{E}-x_{S}\right)\\ y_{E}-y_{S} \end{array}\right]$$ (1)

$$\theta_{\text{arm}}=\mathrm{atan}2\left(\Delta y,\Delta x\right)\times \left(\frac{180}{\pi }\right)$$ (2)

where the subscripts E and S denote the elbow and shoulder landmarks, respectively. Using the angle defined in Equation 2, θ_arm is used as the control variable for horizontal (reach) adjustment, while the lower-arm flexion angle θ_lower (Equation 3) serves as the control variable for vertical (height) adjustment. Each estimated angle is continuously compared with its own predefined ergonomic target range to determine whether workstation adjustment is required, including workpiece height, reach distance, or tilt angle. Dead-zone thresholds are incorporated for each axis to prevent unnecessary robot motion caused by minor posture fluctuations around the target range; the specific target ranges and control laws are detailed in Section 2.4.

An automated Rapid Upper Limb Assessment (RULA) was performed using the full set of skeletal landmarks to evaluate overall ergonomic risk. Joint angles for the upper arm, lower arm, wrist, and neck/trunk were computed from the estimated skeletal landmarks and classified into RULA score bands using a rule-based (if–then) implementation of the original scoring tables (McAtamney and Corlett, 1993). The resulting individual scores were combined using Table A (arm and wrist) and Table B (neck, trunk, and legs), which were then mapped to the final Grand Score via Table C, following the standard RULA scoring procedure. Although RULA is not used as a control input, it provides an objective measure for assessing the ergonomic effectiveness of the proposed framework.

### 2.4 Robot adaptation

The robot adaptation module executes the adjustment decisions generated by the ergonomic decision-making module by generating and sending precise motion commands to the collaborative robot. A closed-loop controller is employed to compute the required velocity commands for workstation adaptation based on two independently estimated posture angles: the upper-arm orientation angle (θ_arm) and the lower-arm flexion angle (θ_lower). The vertical velocity command is computed from the deviation between the estimated lower-arm flexion angle and the target ergonomic angle using the control law shown in Equation 4, where θ_lower is computed as described in Equation 3. The horizontal (reach) velocity command is computed from the upper-arm orientation angle using the control law shown in Equation 5.

The lower-arm flexion angle (θ_lower) was computed as the three-dimensional angle at the elbow joint between the shoulder-to-elbow and elbow-to-wrist vectors, using the (x, y, z) coordinates of the shoulder, elbow, and wrist landmarks estimated by MediaPipe Pose:

$$\theta_{lower}=\arccos\left(\frac{\text{ba}\cdot \text{bc}}{\left|\text{ba}\right|\left|\text{bc}\right|}\right)\times \frac{180}{\pi }$$ (3)

where *ba* = a − b and *bc* = c − b, with a, b, and c denoting the shoulder, elbow, and wrist landmark positions, and b serving as the vertex of the angle at the elbow joint.

The vertical velocity command was computed from the deviation between the lower-arm flexion angle and its ergonomic target range (90°–100°), using a dead-zone with saturated proportional control (Equation 4). To reduce mechanical jerking, both the estimated angle and the resulting velocity command were additionally smoothed using exponential moving average filters prior to being sent to the robot controller.

$$v_{z}=\left\{\begin{array}{cc} 0 & \text{if }90^{\circ }\leq \theta_{lower}\leq 100^{\circ }\\ v_{\max,z}\cdot \frac{\min\left(\theta_{lower}-100^{\circ },\Delta \theta_{\max}\right)}{\Delta \theta_{\max}} & \text{if }\theta_{lower}>100^{\circ }\\ -v_{\max,z}\cdot \frac{\min\left(90^{\circ }-\theta_{lower},\Delta \theta_{\max}\right)}{\Delta \theta_{\max}} & \text{if }\theta_{lower}<90^{\circ } \end{array}\right.$$ (4)

where v_max,z = 0.06 m/s and Δθ_max = 15°.

The horizontal (reach) velocity command was computed from the upper-arm orientation angle using a symmetric dead-zone with saturated linear speed interpolation (Equation 5); no additional smoothing filter was applied to this axis.

$$v_{y}=\left\{\begin{array}{cc} 0 & \text{if}\;\left|\theta_{arm}\right|\leq \theta_{th}\\ \text{sign}\left(\theta_{arm}\right)\cdot \left[v_{\min,y}+\min\left(1,\frac{\left|\theta_{arm}\right|-\theta_{th}}{\theta_{sat}-\theta_{th}}\right)\cdot \left(v_{\max,y}-v_{\min,y}\right)\right] & \text{otherwise} \end{array}\right.$$ (5)

where θ_th = 20° is the dead-zone threshold, θ_sat = 90° is the saturation angle, v_min,y = 0.03 m/s, and v_max, y = 0.06 m/s.

In addition to positional adaptation, the wrist orientation of the collaborative robot was adjusted to maintain a consistent tool inclination during task execution. The wrist angle was not estimated from the operator’s posture; rather, the final wrist joint (Wrist 3, Joint 6) was commanded to a predefined orientation once the operator reached a stable working posture, independent of any measured wrist landmark. This approach minimizes unnecessary wrist rotation while maintaining consistent accessibility of the workpiece.

A predefined wrist orientation of φ_wrist = −55° was selected to maintain a consistent workpiece inclination throughout the task. Since the UR robot controller receives joint commands in radians, this value was converted to q6 = −0.9599 rad, where q6 denotes the target joint angle of Wrist 3 (Joint 6) expressed in radians. During adaptation, the current robot joint configuration, **q**_current_, was first obtained, where **q**_current_ = [q1, q2, q3, q4, q5, q6]. The target joint configuration was then generated according to Equation 6 by replacing only the Wrist 3 joint value:

$$q_{\text{target}}=\left[\begin{array}{cccccc} q_{1}, & q_{2}, & q_{3}, & q_{4}, & q_{5}, & -0.9599 \end{array}\right]$$ (6)

where the first five joint positions were preserved to avoid unnecessary motion of the robot arm, while only the final wrist joint was adjusted to achieve the desired tool orientation.

To ensure safe human–robot interaction, a 3D adaptive safety zone was established around the operator’s torso. The torso central axis unit vector, **u**_axis_, was defined from the shoulder centre (**p**_top_) toward hip centre (**p**_bot_) as given in Equation 7:

$$u_{\text{axis}}=\frac{p_{\text{bot}}-p_{\text{top}}}{\left\|p_{\text{bot}}-p_{\text{top}}\right\|}$$ (7)

Equation 7 normalizes the vector from the shoulder centre to the hip centre to obtain a unit vector representing the central axis of the operator’s torso. This unit vector serves as the reference axis for calculating the perpendicular distance between the robot end-effector and the operator’s torso.

The adaptive safety distance **D**_safety_ was dynamically calculated using Equation 8:

$$D_{\text{safety}}=\max\left(R_{\text{shoulder}},R_{\text{hip}},0.35\right)+0.05m$$ (8)

The resulting safety distance was at least 0.40 m and increased automatically according to the operator’s shoulder or hip dimensions when a larger safety zone was required. The actual distance D_actual_ between the robot end-effector and the torso axis was computed using orthogonal projection. If D_actual_ < D_safety_ and the robot velocity was directed toward the operator (v_y < 0), the velocity command was immediately set to zero. A pseudocode summary of the complete perception–decision–adaptation control loop is provided in the Supplementary Materials.

## 3 Materials and methods

A within-subject experiment was conducted to evaluate the proposed adaptive robotic workstation. Participants performed a collaborative screw-driving task under adaptive and fixed workstation conditions at both near and far working distances.

### 3.1 Participants

The target population comprised working-age adults with no history of musculoskeletal disorders (MSDs). Thirty healthy adults (15 males and 15 females) participated in the study. Participants had a mean age of 42.9 ± 7.7 years and a mean height of 164.0 ± 8.4 cm. Participants were recruited from industrial workers who routinely performed manual tool-handling tasks and were stratified into three height groups (low, medium, and high) to ensure balanced anthropometric representation. None had prior experience working with cobots. All participants were right-handed, had prior experience using a screwdriver, and reported no history of musculoskeletal disorders. Ethical approval for the study was obtained from the Institutional Review Board (KMUTT-IRB-2026/0226/155), and written informed consent was obtained from all participants prior to data collection.

### 3.2 Experimental setup

Figure 2A illustrates the experimental setup, including a schematic of the collaborative screw-driving task, the workspace layout, dimensions, and safety parameters. Each participant completed a standardized screw-driving assembly task using a manual screwdriver under two workstation conditions: (1) a fixed workstation, in which the workpiece position remained unchanged (Figure 2B), and (2) an adaptive workstation, in which the collaborative robot automatically adjusted the workpiece position according to the operator’s upper-limb posture (Figure 2C). The workpiece consisted of a simulated battery module measuring 29 cm in width with multiple screw locations. In the fixed workstation, the workpiece was mounted parallel to the ground, whereas in the adaptive workstation, the workpiece orientation was configured with predefined inclination and lateral tilt angles to improve reachability and visual accessibility. Since all participants were right-handed, the same workpiece orientation was used throughout the experiment. Participants were required to keep their feet planted in a fixed position throughout each trial in both workstation conditions, while natural compensatory movement of the torso and arms (e.g., leaning or reaching) was permitted. This constraint was applied identically across conditions to ensure that observed differences in RULA scores reflect the effect of workstation adaptation rather than differences in permitted participant movement. To minimize learning and order effects, the workstation conditions were randomized and counterbalanced across participants.

**FIGURE 2:**
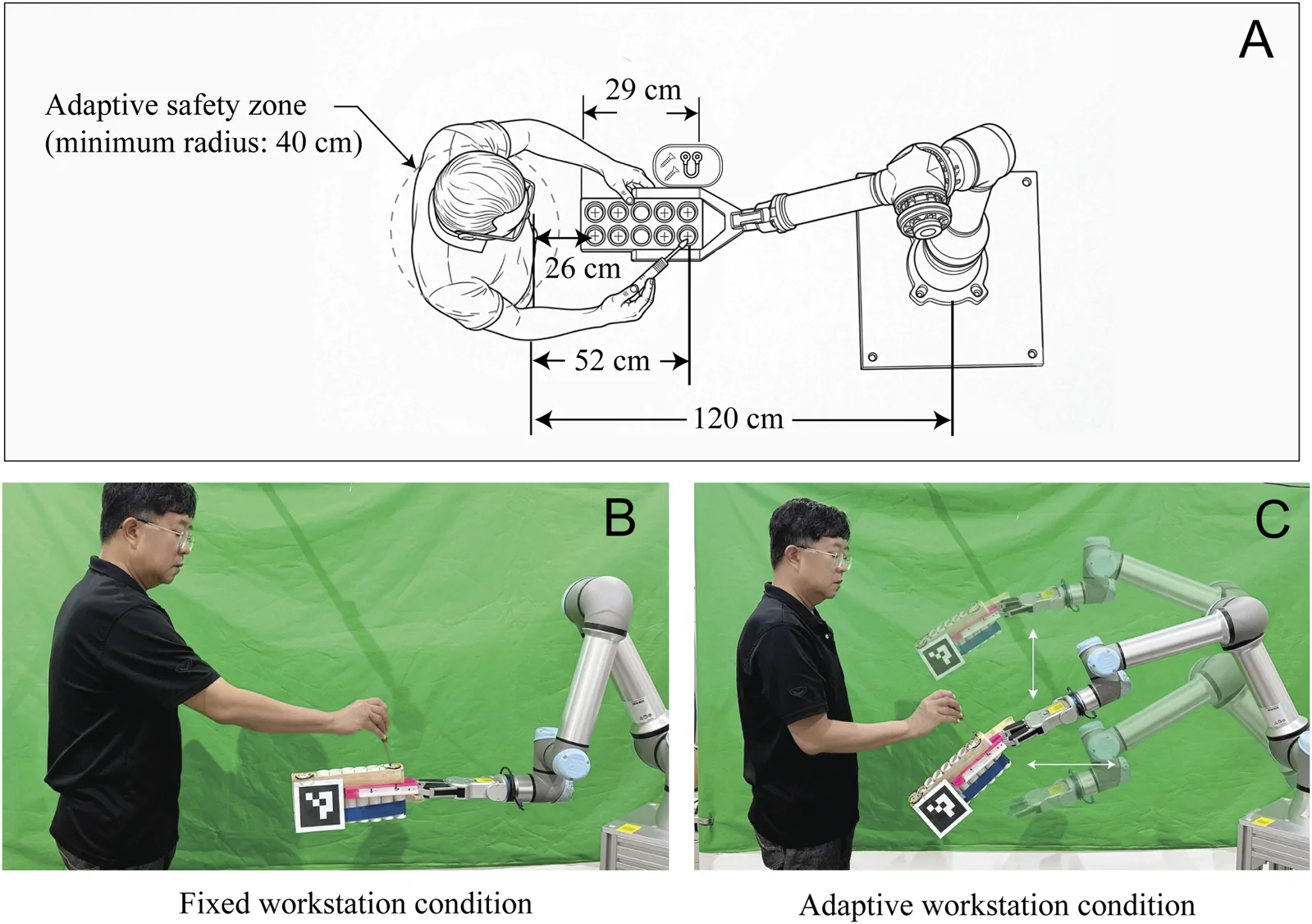
Experimental setup comparing the **(B)** fixed workstation and **(C)** adaptive workstation with active ergonomic positioning. **(A)** Schematic of the workspace dimensions and safety parameters.

### 3.3 Experimental procedure

Before the experiment, participants received an explanation of the study objectives, experimental procedures, and task requirements, followed by a brief familiarization session. Each participant then performed the standardized screw-driving task under both workstation conditions. For each workstation condition, participants completed four screw-driving operations, including two screws positioned at the near reach distance (26 cm from the participant’s anterior torso reference point) and two screws positioned at the far reach distance (52 cm from the participant’s anterior torso reference point). Upper-limb kinematic data were continuously recorded during task execution. The mean completion time for an individual screw-driving task was 55.4 ± 20.1 s, ranging from 43.9 s (fixed workstation, far reach) to 69.5 s (adaptive workstation, near reach) depending on condition.

The adaptive workstation operated according to the following sequence:

**Step 1:** The operator grasped the screwdriver and reached toward the workpiece. The robotic arm simultaneously moved the workpiece closer to reduce the initial reaching distance (Figure 3A).
**Step 2:** The robot continuously monitored the operator’s elbow angle during the reaching phase. When the estimated elbow angle remained within the predefined ergonomic target range (90°–100°) for approximately 1 s, the system confirmed a stable working posture and applied the predefined workpiece inclination of 40° to improve visual accessibility and task interaction (Figure 3B). If the elbow angle did not satisfy this condition, the workpiece inclination remained unchanged.
**Step 3:** The workpiece was subsequently maintained at a predefined lateral orientation of approximately 55° to improve accessibility and support a more neutral wrist posture during screw-driving operations (Figure 3C).
**Step 4:** The operator removed the two proximal (near-side) screws using the screwdriver (Figure 3D).
**Step 6:** The operator then reached toward the distal (far-side) screws. During this phase, the robot continuously adjusted the workpiece position, including height and reach distance, based on the operator’s upper-limb posture to reduce excessive reaching and maintain an ergonomic working posture (Figure 3E).

**FIGURE 3:**
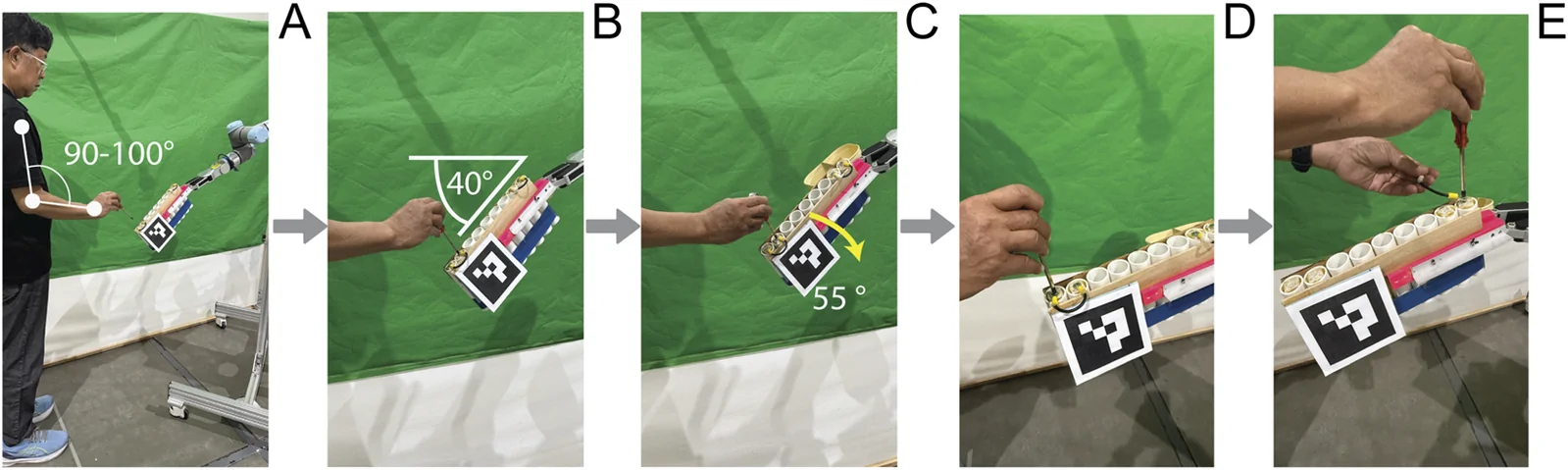
Workflow of the adaptive human–robot collaborative screw-driving task. **(A)** Robot approach. **(B)** Posture verification. **(C)** Workpiece rotation. **(D)** Proximal screw removal. **(E)** Distal screw removal.

After completing each condition, participants completed a 5-point Likert-scale questionnaire evaluating physical comfort, postural load, trust, acceptance, and perceived safety. A 10-min rest period was provided between workstation conditions to minimize fatigue.

### 3.4 Experimental measures and statistical analysis

The experiment employed a 2 × 2 within-subject design with workstation condition (fixed and adaptive) and reach distance (near and far) as the independent variables. The ergonomic performance of the proposed workstation was evaluated using both objective and subjective measures. Objective measures included the RULA score and task completion time. RULA scores were calculated from the upper-limb postures estimated by the vision-based posture perception system described in Section 2.2 and served as the primary measure of ergonomic risk. Because the screw-driving task involved continuous, dynamic movement rather than a single static posture, RULA scores were computed internally at each frame of the vision-based posture perception system (30 fps) throughout each trial. For data logging and subsequent statistical analysis, RULA values were recorded to file at a throttled rate of approximately 5 Hz (mean logging interval = 221.7 ± 73.2 ms). The reported RULA score for each trial was calculated as the time-weighted average of these logged scores across the full task duration, providing a measure of overall ergonomic exposure rather than a single static assessment. This averaging approach was adopted to capture overall postural exposure across the task rather than isolated peak-risk moments, consistent with the continuous nature of the screw-driving activity. Task completion time was defined as the elapsed time from the start of each trial to completion of the screw-driving task. For the adaptive workstation, this duration included the robot repositioning phase before task execution. Descriptive statistics (mean ± standard deviation) were calculated for all outcome measures. The effects of workstation condition and reach distance on RULA scores and task completion time were analysed using two-way repeated-measures analysis of variance (ANOVA). Bonferroni-adjusted *post hoc* comparisons were performed when significant main or interaction effects were observed. Subjective ratings were compared between the fixed and adaptive workstation conditions using paired-samples t-tests and Wilcoxon signed-rank tests. Because the subjective measures were based on ordinal 5-point Likert-scale ratings, and Shapiro–Wilk tests indicated departures from normality for three of the four constructs, the Wilcoxon signed-rank test was used as the primary inferential test for all subjective measures, with paired-samples t-test results reported alongside for comparison. All statistical analyses were performed using Jamovi (Version 2.5.7), with statistical significance set at p < 0.05.

## 4 Results

### 4.1 Ergonomic performance

Table 1 summarizes the descriptive statistics of the RULA scores for each workstation condition and reach distance. Across both reach distances, the adaptive workstation consistently produced lower RULA scores than the fixed workstation, indicating reduced ergonomic risk. The lowest mean RULA score was observed for the adaptive workstation under the near-reaching condition (*M* = 4.47, *SD* = 0.67), whereas the highest mean score occurred for the fixed workstation under the far-reaching condition (*M* = 6.16, *SD* = 0.48).

**TABLE 1:** Descriptive statistics of RULA scores across workstation type and reach distance.

Workstation	Reach distance	Mean RULA	SD
Adaptive	Near	4.47	0.67
Adaptive	Far	4.59	0.70
Fixed	Near	5.68	0.66
Fixed	Far	6.16	0.48

A two-way repeated-measures ANOVA revealed significant main effects of workstation type, *F* (1, 29) = 138.3, *p* < 0.001, η^2^_p_ = 0.827, and reach distance, *F* (1, 29) = 19.5, *p* < 0.001, η^2^_p_ = 0.402 (see Table 2). A significant interaction effect was also observed between workstation type and reach distance, *F* (1, 29) = 14.1, *p* < 0.001, η^2^_p_ = 0.327, indicating that the effect of workstation type depended on the reaching distance.

**TABLE 2:** Results of the two-way repeated-measures ANOVA for RULA scores.

Effect	df	F	p	η^2^p
Workstation type	1,29	138.3	<0.001	0.827
Reach distance	1,29	19.5	<0.001	0.402
Workstation type ✻ reach distance	1,29	14.1	<0.001	0.327

Bonferroni-adjusted pairwise comparisons showed that the adaptive workstation significantly reduced RULA scores compared with the fixed workstation under both the near-reaching (mean difference = −1.259, SE = 0.140, *p* < 0.001) and far-reaching conditions (mean difference = −1.737, SE = 0.099, *p* < 0.001). The reduction in ergonomic risk was greater during the far-reaching task. Furthermore, no significant difference in RULA scores was observed between the near- and far-reaching conditions for the adaptive workstation (*p* = 0.217), whereas the fixed workstation exhibited significantly higher RULA scores during the far-reaching task (mean difference = −0.479, SE = 0.100, *p* < 0.001).

Figure 4 illustrates the mean RULA scores across workstation conditions and reach distances. The adaptive workstation consistently resulted in lower RULA scores than the fixed workstation for both near- and far-reaching tasks. In particular, the difference between workstation conditions became larger during the far-reaching task, where the adaptive workstation maintained a relatively stable ergonomic risk level while the fixed workstation showed an increased RULA score. This pattern corresponds with the significant interaction between workstation condition and reach distance.

**FIGURE 4:**
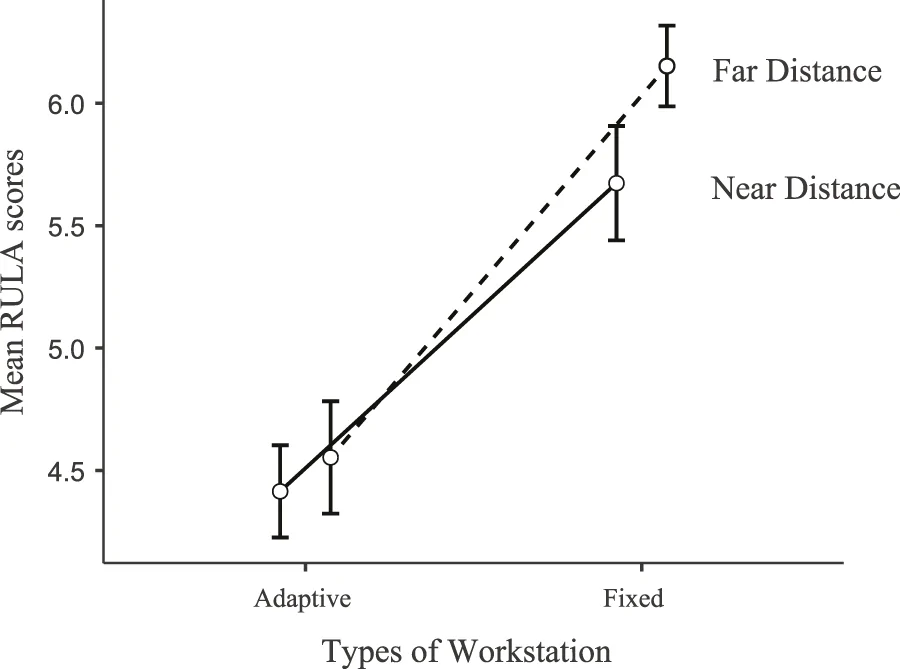
Interaction effect of workstation condition and reach distance on mean RULA scores under adaptive and fixed workstation conditions.

### 4.2 Task completion time

Task completion times for each workstation condition and reach distance are summarized in Table 3. A two-way repeated-measures ANOVA revealed significant main effects of task distance, *F* (1, 29) = 14.64, *p* < 0.001, η^2^_p_ = 0.335, and workstation type, *F* (1, 29) = 39.82, *p* < 0.001, η^2^_p_ = 0.579, indicating that completion time differed across both reach distances and workstation configurations. However, the interaction between task distance and workstation type was not significant, *F* (1, 29) = 1.31, *p* = 0.263, η^2^_p_ = 0.043, suggesting that the effect of workstation type was consistent across reach distances. Descriptively, the adaptive workstation required longer completion times than the fixed workstation for both near-reaching (69.5 ± 24.6 s vs. 50.2 ± 13.6 s) and far-reaching tasks (57.8 ± 19.4 s vs. 43.9 ± 10.7 s).

**TABLE 3:** Two-way repeated-measures ANOVA results for task completion time.

Source	F (1,29)	p-value	η^2^p
Distance	14.64	<0.001	0.335
Workstation	39.82	<0.001	0.579
Distance * workstation	1.31	0.263	0.043

### 4.3 User experience

Participants reported significantly greater physical comfort when using the adaptive workstation than the fixed workstation (Table 4), t (29) = −4.93, p < 0.001, d = 0.90 (Wilcoxon V = 19.00, p < 0.001, r = −0.892). The adaptive workstation also significantly reduced perceived postural load, t (29) = −7.30, p < 0.001, d = 1.33 (V = 4.00, p < 0.001, r = −0.977), and increased trust and acceptance, t (29) = −4.26, p < 0.001, d = 0.78 (V = 17.00, p < 0.001, r = −0.853). In contrast, perceived safety did not differ significantly between workstation conditions under either test, t (29) = −0.78, p = 0.445, d = 0.14 (V = 51.50, p = 0.241, r = −0.327). Given the ordinal nature of the Likert-scale ratings, non-parametric Wilcoxon signed-rank tests were conducted alongside paired t-tests; results were consistent across both approaches for all four constructs, supporting the robustness of these findings.

**TABLE 4:** Comparison of user satisfaction construct scores between fixed and adaptive workstation conditions.

Construct	Fixed, M(SD)	Adaptive M(SD)	t	p	Cohen’s d	Wilcoxon V	p	Rank-biserialr
Physical comfort	3.45 (1.02)	4.41 (0.63)	−4.93	<0.001	0.90	19.00	<0.001	−0.892
Postural load	2.97 (0.67)	4.46 (0.72)	−7.30	<0.001	1.33	4.00	<0.001	−0.977
Perceived safety	4.15 (0.95)	4.30 (0.90)	−0.78	0.445	0.14	51.50	0.241	−0.327
Trust & acceptance	3.93 (0.87)	4.52 (0.62)	−4.26	<0.001	0.78	17.00	<0.001	−0.853

### 4.4 Anthropometric adaptability

To evaluate the anthropometric adaptability of the proposed workstation, linear regression analyses were conducted to examine whether participant height influenced ergonomic performance under each workstation condition (Table 5).

**TABLE 5:** Linear regression analysis of participant height and mean RULA score under each workstation condition.

Condition	Slope (B)	*R* ^2^	p
Adaptive workstation mean	0.0197	0.089	0.108
Fixed workstation mean	0.0229	0.149	0.035

For the fixed workstation, participant height significantly predicted mean RULA score (B = 0.0229, *p* = 0.035, *R* ^2^ = 0.149), indicating that taller participants generally experienced higher ergonomic risk than shorter participants. This finding suggests that a fixed workstation was less capable of accommodating anthropometric differences, requiring users to adapt their posture to the workstation. In contrast, participant height was not a significant predictor of mean RULA score for the adaptive workstation (B = 0.0197, *p* = 0.108, *R* ^2^ = 0.089). Although a positive trend remained, the relationship was weaker and did not reach statistical significance, suggesting that the adaptive workstation reduced the influence of participant height on ergonomic performance.

Figure 5 illustrates the linear relationship between participant height and mean RULA score under both workstation conditions. The regression line for the fixed workstation exhibited a steeper positive slope than that for the adaptive workstation, indicating a stronger relationship between participant height and mean RULA score. This visual pattern is consistent with the regression analysis presented in Table 5, where participant height was a significant predictor of mean RULA score for the fixed workstation but not for the adaptive workstation. Two individual data points in the adaptive workstation condition (participant heights of approximately 165 cm and 180 cm) exhibited noticeably higher RULA scores than the surrounding data. Post-hoc review of the corresponding trial recordings indicated that these two participants tended to retain habitual movement patterns from their typical working posture, rather than fully adjusting to the robot-repositioned workpiece, resulting in less ergonomically favorable postures than the adaptive system was designed to support. This suggests that individual behavioral habits may, in some cases, offset the ergonomic benefit provided by workstation adaptation.

**FIGURE 5:**
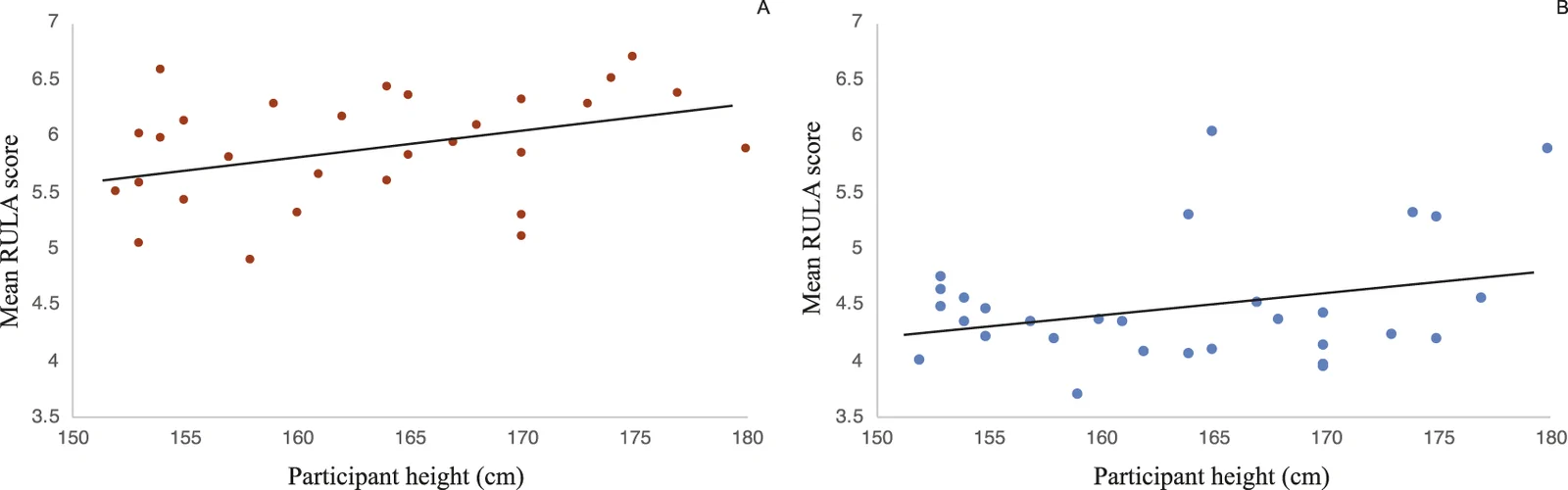
Effect of participant height on mean RULA score under **(A)** fixed and **(B)** adaptive workstation conditions.

## 5 Discussion

The present study demonstrates that integrating human posture perception into robot control enables a transition from conventional fixed human–robot interaction toward adaptive human–robot collaboration, addressing a gap in real-time, posture-driven workspace adaptation (Cardoso et al., 2021; Ajoudani et al., 2018; Lorenzini et al., 2023).

In this study, the adaptive workstation continuously adjusted the robot end-effector position based on the operator’s posture, resulting in significantly lower RULA scores compared with the fixed workstation. This finding is consistent with previous research demonstrating that adaptable workstations can improve ergonomic conditions in human–robot collaborative environments by modifying the physical workspace according to operator requirements (Kim et al., 2019). Unlike static ergonomic interventions, the proposed system enables the robot to actively participate in maintaining appropriate working postures through closed-loop interaction. This concept is consistent with recent studies emphasizing that collaborative robots should actively adapt their behavior based on human ergonomic states rather than merely optimizing immediate task performance (Zolotas et al., 2024). More recently, human–robot collaboration (HRC) frameworks have further integrated real-time ergonomic assessment into robot decision-making, enabling adaptive robot behavior based on the operator’s physical condition rather than predefined task execution (Iodice et al., 2025).

The ergonomic benefit of adaptation was greater under far-reaching conditions than near-reaching conditions, consistent with the significant interaction effect reported above. This finding suggests that adaptive robot behavior may be particularly valuable for tasks involving greater reach distances, where postural demands on the upper limb are inherently higher. Previous ergonomic HRC studies have emphasized that reaching distance and workspace configuration are important determinants of upper-limb workload during collaborative tasks (Keshvarparast et al., 2024). Therefore, the ability of the robot to compensate for increased reaching requirements represents an important step toward more human-centered collaborative systems.

Although the adaptive workstation improved ergonomic performance, it required longer task completion times compared with the fixed workstation. This result highlights an important trade-off in adaptive human–robot interaction, where additional robot adjustments may increase execution time while providing improved physical support. Unlike conventional workstations, adaptive robotic systems require users to interact with a dynamic workspace rather than follow a fixed configuration. For the adaptive condition, this duration also included the robot repositioning phase itself, and participants were required to interpret and compensate for changes in robot height and reach distance to maintain an appropriate working posture—for example, adjusting elbow position in the opposite direction of robot motion. Human–robot interaction studies have emphasized that effective collaboration requires users to develop an accurate mental model of robot behavior (Goodrich and Schultz, 2007; Sheridan, 2016), and this within-trial interpretive demand offers a plausible mechanistic explanation for the increased completion time independent of prior robot experience. However, because each participant completed only a single trial per condition, this study cannot determine whether the additional completion time reflects a stable interaction cost or diminishes with repeated exposure to the adaptive system. The lack of longitudinal or repeated-session data is a limitation of the present study, and future work should investigate whether task efficiency improves as users become more familiar with the adaptive workstation’s behavior. Previous research has highlighted that successful human–robot collaboration involves adaptation from both the robot and the human partner (Ajoudani et al., 2018).

A further limitation concerns the fixed foot position imposed throughout each trial. This constraint was necessary to preserve the near- and far-reach distance manipulation, which was defined relative to the participant’s own body position; unrestricted repositioning would have allowed participants to compensate for reach demands independently of the robot, confounding the intended comparison. In real assembly settings, however, workers can often step or reposition freely, which may itself serve as a natural ergonomic strategy—particularly by reducing trunk flexion, a key contributor to RULA scoring. Consequently, the ergonomic advantage of the adaptive workstation observed here may be more pronounced than in settings where workers can freely reposition, and future work should evaluate the system under less constrained movement conditions to assess its ecological validity. Notably, many real-world collaborative workcells similarly constrain operator footprint for safety or fixture-related reasons, so the present findings remain directly relevant to that class of deployment scenarios.

The subjective evaluation demonstrated that the adaptive workstation improved physical comfort, reduced perceived postural load, and increased trust and acceptance, while perceived safety remained comparable to the fixed condition. This finding suggests that the adaptive behavior of the robot provided ergonomic benefits without negatively affecting users’ safety perception. Although collision avoidance mechanisms were implemented to prevent unsafe interactions, qualitative feedback from the open-ended questionnaire indicated that some participants remained concerned when the robot moved closer to their body or exhibited unexpected movements. This finding indicates that physical safety mechanisms alone may not fully determine human perception of safety. Previous Human-Robot Interaction (HRI) studies have emphasized that effective collaboration depends not only on technical capability but also on the fluency and coordination of HRI (Hoffman, 2019; Forlini et al., 2023). Therefore, future adaptive robotic systems should consider not only physical safety constraints but also mechanisms that improve robot motion transparency and predictability. Providing clearer feedback about robot intention and operational state may further enhance user confidence while maintaining the ergonomic advantages of adaptive human–robot collaboration.

The present study also demonstrates that the adaptive workstation reduced the influence of anthropometric variability on ergonomic outcomes, consistent with the regression findings reported above (Section 4.4). Recent studies in human–robot collaboration have emphasized the transition toward human-centered manufacturing, where robotic systems should accommodate individual worker characteristics through adaptive and personalized workplace configurations (Lorenzini et al., 2023; Ojsteršek et al., 2024; Panagou et al., 2024). Extending this direction, the present study demonstrates that real-time human posture perception can enable the robot to dynamically modify the physical workspace and provide more consistent ergonomic conditions across users with different anthropometric characteristics. This capability represents an important step toward personalized and human-centered collaborative robotic systems.

Future adaptive systems should advance from posture-based control toward predictive human perception. While elbow posture was an effective control variable in this study, operators may adopt compensatory postures depending on task requirements, such as raising the workpiece to improve visual accessibility while increasing elbow elevation. Therefore, future robots should consider operator intention and task context to provide more comprehensive and personalized ergonomic adaptation.

## 6 Conclusion

This study developed and evaluated a vision-guided adaptive robotic workstation that dynamically adjusted the robot workspace according to operator posture during human–robot collaboration. The experimental results demonstrated that the adaptive workstation significantly reduced ergonomic risk compared with the fixed workstation, particularly during far-reaching tasks where ergonomic demands were more pronounced. The proposed system also reduced the influence of anthropometric variability on ergonomic outcomes. Unlike the fixed workstation, where participant height significantly affected RULA scores, the adaptive workstation provided more consistent ergonomic conditions across operators with different heights. In addition, participants reported improved physical comfort, lower perceived postural load, and higher trust and acceptance, while perceived safety remained comparable to the fixed condition. These findings highlight the potential of posture-based adaptive robots to move beyond conventional fixed workspaces toward more human-centered collaborative systems. Although the adaptive approach introduced additional task completion time, the results suggest that improved ergonomic support and user experience can be achieved through human-aware robot adaptation. Future research should investigate predictive and context-aware adaptation strategies that incorporate task requirements and operator intentions to further enhance efficiency and personalized human–robot collaboration.

## Data Availability

The datasets presented in this study can be found in online repositories. The names of the repository/repositories and accession number(s) can be found in the article/Supplementary Material.

## Supplementary material

SUPPLEMENTARY DATA SHEET S1
Participant characteristics and subjective evaluation data under fixed and adaptive workstation conditions.

SUPPLEMENTARY DATA SHEET S2
RULA scores under fixed and adaptive workstation conditions at near and far working positions.

SUPPLEMENTARY FILE S1
Pseudocode summary of the real-time control loop.
