# Supplementary material for: A vision-based closed-loop framework for adaptive ergonomic human-robot collaboration
Source: Front Robot AI. 2026 Oct 14;13:1948863. doi: 10.3389/frobt.2026.1948863 (PMC13713420; doi:10.3389/frobt.2026.1948863)
Supplement: Supplementary file 1 [file Supplementaryfile1.pdf]

Algorithm S1. Pseudocode summary of the real-time control loop, reflecting the per-frame execution order (which may differ from the expository order presented in Sections 2.3–2.4 for clarity).

```
ALGORITHM: Real-Time Ergonomic Workstation Adaptation Loop
INPUT:  Front-facing camera stream (body posture, 30 fps)
        Top-mounted camera stream (hand/wrist, 30 fps)
OUTPUT: Robot velocity commands (v_y, v_z)
        Wrist joint command (q_target), triggered once per stable posture
LOOP (executed continuously during task execution):

    1. PERCEPTION
       {shoulder, elbow, wrist} ← MediaPipe Pose(front-camera frame)
       {hand landmarks} ← MediaPipe Hands(top-camera frame)

       (Δx, Δy) ← vector components from shoulder, elbow         [Eq. 1]
       θ_arm ← atan2(Δy, Δx) × (180/π)                            [Eq. 2]
       θ_lower ← elbow-joint angle from shoulder–elbow–wrist      [Eq. 3]
    2. ROBOT ADAPTATION (velocity calculation)
       v_z ← dead-zone/saturation control law on θ_lower           [Eq. 4]
       v_y ← dead-zone/saturation control law on θ_arm             [Eq. 5]
    3. SAFETY CHECK (every iteration, independent of adjustment need)
       u_axis ← normalize(p_bot − p_top)                          [Eq. 7]
       D_safety ← max(R_shoulder, R_hip, 0.35) + 0.05              [Eq. 8]
       D_actual ← orthogonal_distance(robot_end_effector, torso_axis)
       IF D_actual < D_safety AND v_y < 0:
           v_y ← 0    // override: block motion toward operator
    4. STABILITY / WRIST-TRIGGER CHECK
       IF θ_arm and θ_lower remain within their ergonomic target
          ranges for a sustained interval:
           q_target ← [q1, q2, q3, q4, q5, ϕ_wrist → rad]          [Eq. 6]
           SEND q_target to robot controller   // wrist move replaces
                                                 // velocity send this cycle
       ELSE:
           SEND (v_y, v_z) to robot controller
    5. ERGONOMIC ASSESSMENT (logged, not used as control input)
       RULA_score ← rule-based classification(all skeletal landmarks:
                     upper arm, lower arm, wrist, neck, trunk, legs)
    6. RE-ESTIMATE posture and repeat from Step 1

END LOOP
```
